# Online Communities as a Driver for Patient Empowerment: Systematic Review

**DOI:** 10.2196/19910

**Published:** 2021-02-09

**Authors:** Victoria Johansson, Anna Sigridur Islind, Tomas Lindroth, Eva Angenete, Martin Gellerstedt

**Affiliations:** 1 University West School of Business, Economics and IT, SE-461 86 Trollhättan Sweden; 2 School of Computer Science Reykjavik University Reykjavik Iceland; 3 Department of Applied IT University of Gothenburg Gothenburg Sweden; 4 Department of Surgery, SSORG - Scandinavian Surgical Outcomes Research Group Institute of Clinical Sciences, Sahlgrenska Academy University of Gothenburg Gothenburg Sweden; 5 Region Västra Götaland, Sahlgrenska University Hospital/Östra Department of Surgery Gothenburg Sweden; 6 School of Health Sciences University of Skövde Skövde Sweden

**Keywords:** patient empowerment, online community, person-centered care, eHealth, systematic review

## Abstract

**Background:**

The use of online resources has changed how people manage health care processes. Patients seek information about health conditions, guidance in treatment, and support from peers online, complementary to traditional health care trajectories. Online communities have the potential to contribute to the quality of care by increasing patient empowerment; however, there is a gap in research regarding in what way online communities contribute to patient empowerment.

**Objective:**

We synthesized research regarding how online communities contribute to patient empowerment to address the research question “In what ways can participation in online communities support patient empowerment?” by studying how patient empowerment is operationalized in different studies. The definition of patient empowerment used in this paper is enablement for people to develop mastery over actions and control over decisions that influence their lives. The mastery is both through processes and outcomes of the development.

**Methods:**

A systematic review was conducted by searching in the following databases: Scopus, ACM Digital Library, EBSCO (CINAHL and MEDLINE), PubMed, and Web of Science. In total, there were 1187 papers after excluding duplicates, and through selection processes using an analytical framework with definitions of patient empowerment and related concepts, 33 peer-reviewed papers were included.

**Results:**

Findings indicated that online communities support patient empowerment both as a process and as outcomes of these processes. Additionally, it was seen as a complement to traditional health care and encouragement for health care professionals to have a more positive attitude toward patients’ usage. There was a mix between deductive (19/33, 58%), inductive (11/33, 33%), and a mixed approach (3/33, 9%) of studying patient empowerment in various forms. The online communities in most papers (21/33, 64%) were well-established and represented patients’ initiatives.

**Conclusions:**

There is a need to include professionals' perspectives regarding how health care can embrace patient empowerment through online communities. This systematic review's main contribution is the proposal of a new framework and conceptualization of how patient empowerment in online communities can be understood from different hierarchical levels.

## Introduction

### Background

When a person faces a difficult situation, for example, when receiving a medical diagnosis, a fear of not being able to control the outcome—feeling disempowered—is a natural response [1]. Up until recently, health care professionals have been the primary resource for helping patients regain empowerment by finding suitable treatment and giving recommendations and support.

### Potential of Online Communities and Patient Empowerment

In parallel to efforts provided by health care, now more than ever, patients use the internet and online communities as complementary health care resources. In a study of the US population, it was found that 80% of internet users (74% of the population) looked for health-related issues, and 18% had gone online to find peers with similar health concerns. This fundamentally changes how people manage their care process, as patients are able to seek guidance, experiences, and support from peers as complementary resources to manage condition of illness and potential posttreatment with the aim of returning to the new normal self [2]. A review [3] found that previous studies have shown that patients use online communities because they experience or believe that health care professionals filter information; are unaware of the latest research; and lack the capability of showing empathy. Thus, online communities serve as supporting resources to increase information and emotional support. Another argument is to get the first-hand experience as a complement to health care expertise, which may help patients translate recommendations and instructions into daily self-care strategies. This could expand patients’ knowledge regarding health conditions and treatments while also helping them find emotional and social support [4], thereby increasing empowerment.

Patient empowerment refers to processes and outcomes at both individual and group level that enable people to develop mastery over actions and control over decisions that influence their lives [5-7]. Patient empowerment could be regarded as being complementary to person-centered care [8,9]. Person-centered care focuses on designing and delivering individualized care, while patient empowerment focuses on a modified relationship between patients and health care professionals that enables patient-driven and patient-centered care [10]. Research indicates that patient-centered approaches are usually more cost-effective [11-13]. Common ground in both concepts is more engaged and informed patients, that is, more empowered patients.

### Difficulties in Online Community Research

Unarguably, the use of online communities has potential, given the right conditions, to be beneficial for patients. The aggregated knowledge found in online communities could serve as a tool for professionals and the whole health care system for quality improvement in health service delivery [14-16]. In this way, online communities could be a contributor to change in health care. However, evidence of efficacy is equivocal with varying results, and comprehensive reviews [3,17-19] show no evidence of harm, but no strong evidence of efficacy either. The same reviews [3,17-19] reported that many of their included studies had methodological weaknesses. Additionally, recent reviews [3,20] reported difficulties in making comparisons between studies due to methodological problems and lack of analytical frameworks [3,20]. Furthermore, benefits are frequently discussed in relation to individual patients. Other factors includes the various challenges posed by usability and sociability of online communities [21-23].

### Objective

Since difficulties in comparing of methodology and efficacy have already been illustrated, this was not the aim of this review. A part of the methodological problem may be lack of a well-established definition of patient empowerment or comprehensive framework related to different levels of the empowerment concept on an individual and collective level in relation to online communities. Therefore, the primary objective for this systematic review was to clarify in which ways participation in online communities can support patient empowerment. This was done by studying how patient empowerment in online communities has been operationalized in different studies.

## Methods

### Information Sources and Eligibility Criteria

The structure of the review followed principles of PRISMA (Preferred Reporting Items for Systematic Reviews and Meta-Analyses) [24]. Specific principles that were followed in the manuscript are presented in Multimedia Appendix 1. Complementary resources that guided the structure were inspired by recently published systematic reviews in the Journal of Medical Internet Research [20,25-31].

For this systematic review, Scopus, ACM Digital Library, EBSCO (CINAHL and MEDLINE), PubMed, and Web of Science were searched. We defined inclusion and exclusion criteria that did not depend on time limitation or a particular research field to receive a high variety of papers and see potential differences or similarities regarding operationalization and definitions of empowerment (Multimedia Appendix 2). Additionally, this decision was made since both patient empowerment and online communities have been researched in a variety of research fields and used different notions. For instance, online community, a notion more recently used based on the phenomenon it refers to for people communicating in so-called internet forums, started in the late 1970s [32].

### Search Strategy

**Figure 1:**
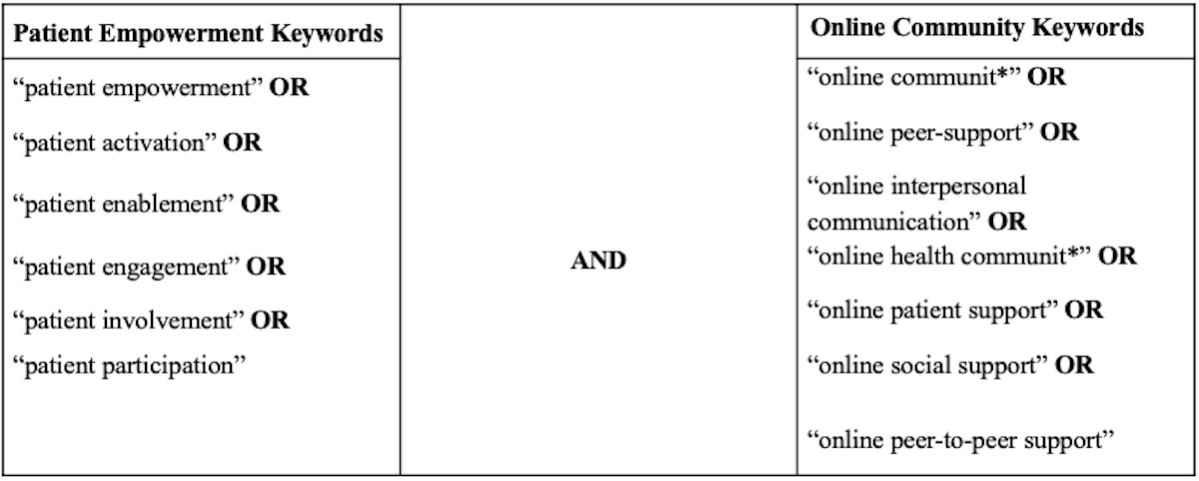
Main search strategy.

Searches in all databases were performed on January 17, 2019 by the first 3 authors. In order to capture relevant papers, 2 search strings were used: (1) patient empowerment and related concepts, and (2) online communities and related concepts. These 2 search strings were combined and used as main search strategy (Figure 1, Multimedia Appendix 3). These 2 search strings included words with a similar meaning or used interchangeably with patient empowerment and online communities. Through this search strategy, we found 1187 references after removing duplicates.

#### Construction of Search Strings

For the patient empowerment search string, we came to rely on the work of defining patient empowerment and related concepts by [5,6] since their work had the purpose of finding a consensus definition of patient empowerment based on previously published research, wherein patient empowerment is described as an umbrella concept—the related concepts (Figure 1) are part of what is considered to be the main definition that the concept entails. Therefore, we did a compilation framework of these definitions of patient empowerment through the related concepts, which was later used as an analytical framework (Multimedia Appendix 4). For the online community search string, all authors did brainstorming sessions together, and the search string was constructed in relation to the inclusion criteria of the Online Community Perspective (Table S1, Multimedia Appendix 2) and through pilot searches of keywords and suggestions of keywords in the selected databases. The combination of the 2, resulted in the final selection of keywords (Figure 1).

### Selection Process

#### Overview

The selection process was conducted by the first 3 authors, in 2 phases. In phase 1, titles, abstracts, and keywords were screened. Phase 2 involved in-depth reading of full texts. The inclusion and exclusion criteria were applied in both phases (Table S1 and Table S2, Multimedia Appendix 2).

#### First Selection Process

**Figure 2:**
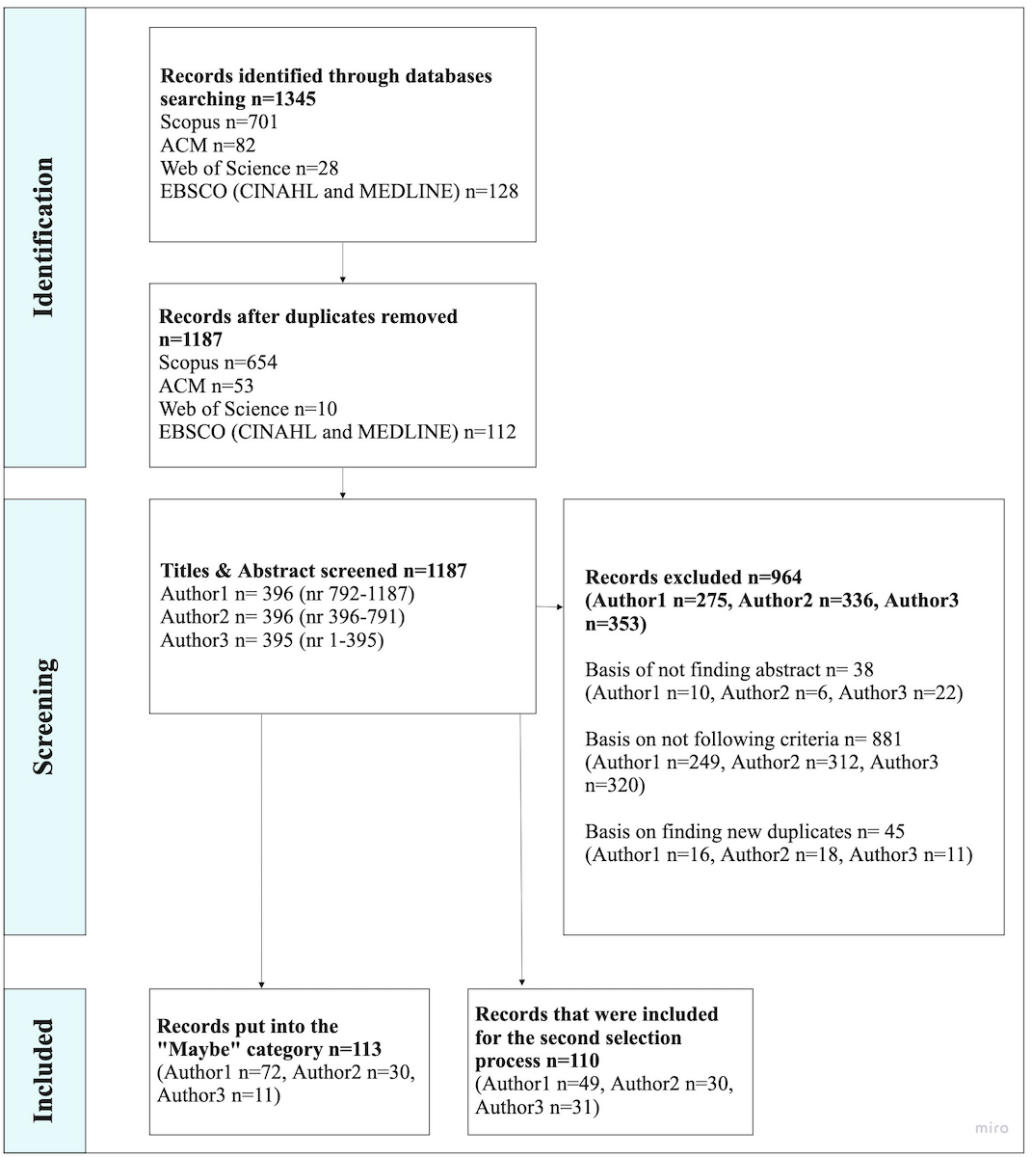
First selection process.

After the first selection process, 223 papers were included (Figure 2). As seen in Figure 2, there was a category named *Maybe*, containing papers for which uncertainty existed regarding inclusion criteria based on only the content of the abstract. Therefore, these papers, alongside the papers with abstracts meeting the inclusion criteria from the first selection process moved forward to the second selection phase.

#### Second Selection Process

**Figure 3:**
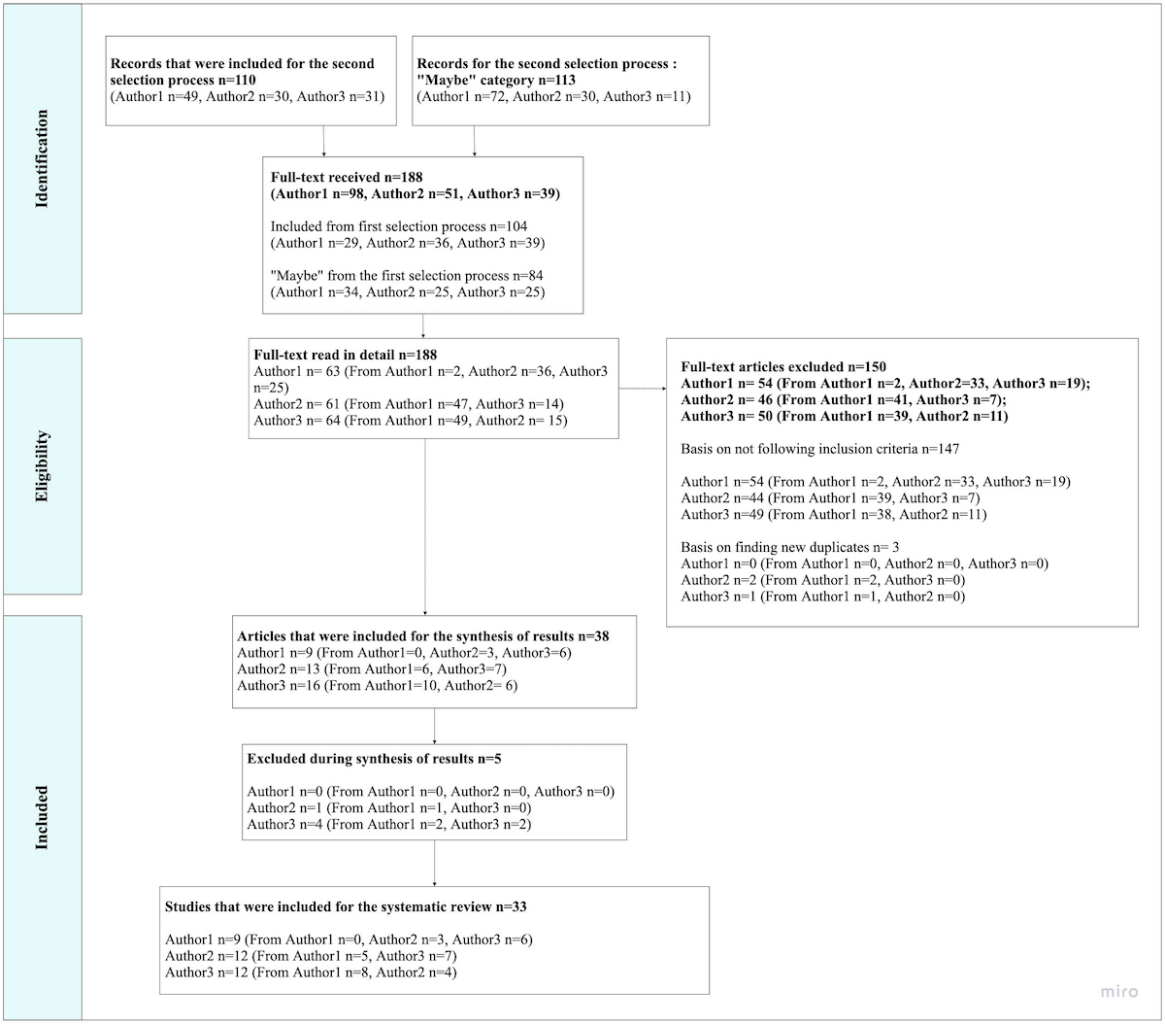
Second selection process.

Papers included in the second phase were assigned to a different screener than the original screener to ensure intercoder reliability (Figure 3, Multimedia Appendix 5). This phase resulted in a final selection of 33 papers. Papers were excluded based on (1) not finding the full text; (2) not meeting the inclusion criteria; (3) in the process of conducting the synthesis of results; (4) meeting the exclusion criteria or it was unclear how, for instance, patient empowerment was evidenced. For the third exclusion reason, after having finalized the second process, we reread each paper multiple times to identify how each paper met the inclusion criteria. Therefore, we discovered that some of the papers that had been included met our exclusion criteria regarding how patient empowerment was evidenced, creating difficulty in remaining neutral to objectively see the characteristics of the paper, without enforcing our own interpretation of how it could answer the research question, and if included, would go against the purpose of conducting systematic literature reviews [33-35] (Table S2, Multimedia Appendix 2).

### Synthesis of Results and Analytical Framework

Two synthesis matrixes were designed prior to analysis to organize potential findings and identify factors involved in these findings [34,36]. The first involved themes of structure and format of each selected paper, that is, author, year, title, format of article, method, and type of online community. The second matrix contained analytical themes that were structured according to frameworks that contained definitions of patient empowerment developed by [5,6] (Multimedia Appendix 4). The analytical framework in the second matrix functioned as a guide to complete the final selection processes and was also used to guide the analysis of results. Each of the 33 papers included were thereby reported in both matrixes and completed with summaries. Finally, all authors were involved in discussion of the synthesis of the results.

## Results

### Overview

The results are divided into 2 parts:

1. Main characteristics: the systematic summarization of selected papers, presentation of main characteristics, that is, type and initiation of online communities, and approach of studying patient empowerment
2. How online communities support patient empowerment: a synthesis of findings in relation to the research question

### Main Characteristics

#### Papers

The papers included in this systematic review were published between 2000 and 2018. Specific details regarding year of publication, journal, methodology, and other related characteristics are presented in Multimedia Appendix 6.

#### Type and Initiation of Online Communities

In the selection, 21 out of 33 papers (64%) presented an online community that was an established community [37-57]; in 7 out of 33 papers (21%), the community type was undefined [58-64], and in 5 papers (15%), the researchers had designed their own community [65-69]. 

#### Established and Undefined Online Community Papers

The established communities targeted specific diagnoses, were communities that were available 24/7, and had many visitors or members over time. The communities existed before being studied (Multimedia Appendix 6). In 2 of these studies, the community was presented as a built-in community through social media, for example, Facebook [40,47]. There were established communities that did not specify the platform, merely that they enabled peer support (3/33, 9%) [46,50,54], while others presented well-established online communities for a specific diagnosis (11/33, 33%) [37-39,41-43,52,54-57]. Additionally, the established online communities were based on patients’ initiatives, both regarding creating (6/33, 18%) [40,43-45,52,57], maintaining and moderating (5/33, 15%) [40,43-45,52], or using the community (21/33, 64%) [37-57].

The papers that had undefined communities mainly aimed to achieve understanding of patient narratives and general experiences from using online communities, instead of the technology in particular. Thus, in 5 of the 7 papers (15%), the aim was to map different behaviors or reasons for use in order to understand the respondents’ levels of patient empowerment [59,61-64], while in 3 out of 7 (9%), the aim was to understand which factors contributed to patient empowerment when using online communities [58,60,64].

#### Involvement of Health Care Professionals

The papers that represented either established or undefined online communities (28/33, 85%) did not discuss or analyze involvement of health care professionals. If health care professionals were mentioned, it was merely to explain why patients used the community (24/33, 73%) [37,39,40,43-62,64], if patients chose to share experiences of use (5/33, 15%) [45,48,49,58,59], or professionals contributed to content in various forms (5/33, 15%), but it was not elaborated on how or if this content had an effect on patients [45,49,53,54,56].

#### Designed Community and Involvement of Health Care Professionals

In the papers that reported having designed online communities, the format was either a web platform [65], online forum [66], own software [68], a list server that created email threads [69], or an e-recovery portal containing online community functionalities [67]. The main function of all designed communities was patient-peer forums. Other functionalities presented were password protection (3/33, 9%) [65,67,68], ability to individually contact health care professionals and design care plans (1/33, 3%) [67], or moderated discussion boards in various formats that was led by researchers (5/33, 15%) [65-69].

In 3 out of 5 papers (3/33, 9%), initiation of use was through joint consultation with health care professionals [65,67,68]. This meant that patients were recruited or recommended by their health care professional in order to participate in a specific online community and study. One of these 3 papers [67] had the health care professionals as part of the study’s results.

#### Studying Patient Empowerment

Of 33 papers, 22 (70%) focused on studying patient empowerment explicitly (Table 1). The focus referred to either a deductive (12/33, 36%) [37-39,47,50-52,55,57,61,65,69] or inductive approach (13/33, 39%) [40,43,46-49,54,57-60,66,67], or a mix thereof. Papers with an inductive approach often revealed patient empowerment as conclusions of thematic analysis or through discussion of findings. Papers with a deductive approach relied on definitions, research, and measurement scales developed by, for example, van Uden-Kraan et al [38,39,70] (7/33, 21%) [37-39,50,51,55,57], Zimmerman [7] (2/33, 6%) [51,53], Spreitzer [71] (2/33, 6%) [47,65], and Barak et al [72] (3/33, 9%) [50,51,57].

The 10 remaining papers (10/33, 30%) did not use patient empowerment explicitly but studied related concepts that are either part of the related concepts presented in the analytical framework (Multimedia Appendix 4) or are related concepts, that is, not explicitly defined in the analytical framework (Table 1). These concepts are presented in Table 1. However, the common denominator of these papers was that they studied specific concepts in a deductive way, thus used relevant measurements and analytical frameworks from previous research of the concept in question.

**Table 1:** Papers that studied patient empowerment or other related concepts.

Characteristic		Established online community		Undefined online community		Designed online community	
		Papers, n (%)	References	Papers, n (%)	References	Papers, n (%)	References
Total papers (N=33)^a^		21 (64)	[37-57]	7 (21)	[58-64]	5 (15)	[65-69]
**Concept studied**							
	Patient empowerment	15 (45)	[37-40,43,46-52,54,55,57]	4 (12)	[58-61]	4 (12)	[65-67,69]
	Patient activation	1 (3)	[45]	0 (0)	N/A^b^	0 (0)	N/A
	Patient engagement	0 (0)	N/A	0 (0)	N/A	1 (3)	[68]
	Adherence to treatment	2 (6)	[44,56]	1 (3)	[64]	0 (0)	N/A
	Self-reappraisal	0 (0)	N/A	1 (3)	[63]	0 (0)	N/A
	Self-efficacy	1 (3)	[56]	0 (0)	N/A	0 (0)	N/A
	Cyber-informational and decisional empowerment	0 (0)	N/A	1 (3)	[62]	0 (0)	N/A
	Intrapersonal and interactional aspect of psychological empowerment	1 (3)	[53]	0 (0)	N/A	0 (0)	N/A
	Individual and collective empowerment	0 (0)	N/A	1 (3)	[64]	0 (0)	N/A
	Well-being	2 (6)	[41,42]	0 (0)	N/A	0 (0)	N/A
	Emotional coping	1 (3)	[41]	0 (0)	N/A	0 (0)	N/A

^a^All percentages refer to the total number of papers, N=33.

^b^N/A: not applicable.

### How Online Communities Support Patient Empowerment

#### Framework

This part will follow the analytical framework structure of presenting different concepts related to patient empowerment: patient enablement, activation, engagement, involvement, and participation (Multimedia Appendix 4).

#### Patient Enablement

Patient enablement is usually presented as the starting phase of becoming empowered and is defined as (1) the possibilities and prerequisites that health care gives the patient to self-manage their own health condition [5] or (2) the patient’s confidence in the ability to improve management of condition or the relationship with health care professionals [6].

#### Established and Undefined Online Communities

For established and unspecified online communities, patient enablement was in 25 out of 33 papers (75%) analyzed as the prerequisite that an online community had for the patient to become engaged and activated in managing diagnosis [37-52,54,55,57-60,62-64]. The prerequisites were often related to patients’ confidence in their own abilities to improve their health conditions or relationships with health care professionals. Patient confidence was analyzed as the context behind the use of online communities, which was divided into (1) becoming better informed, for example, about coping with different treatment alternatives, in order to become more involved in decision-making processes during consultation; (2) coping with the emotional burden of diagnosis in everyday life, by reading and writing content or networking with others with shared experiences; or (3) absorbing and reflecting on information that was perceived as missing or not fully elaborated on during health care consultations (Table 2).

**Table 2:** The direction of context behind patient confidence in selected references.

Characteristic		Established online community		Undefined online community			Designed online community		
		Papers, n (%)	References		Papers, n (%)	References		Papers, n (%)	References
Total papers (N=33)^a^		21 (64)	[37-57]		7 (21)	[58-64]		5 (15)	[65-69]
**Context behind patient confidence**									
	To become better informed	17 (52)	[37-40,43-49,51,53-57]		4 (12)	[58,60,61,64]		4 (12)	[65-68]
	Coping with emotional burden and networking with peers	17 (52)	[37,40-49,51,53-57]		4 (12)	[58,60,61,64]		5 (15)	[65-69]
	Absorb and reflect on information given by health care	15 (45)	[37,40,43-49,51,53-57]		4 (12)	[58,60,61,64]		2 (6)	[65,67]

^a^All percentages refer to the total number of papers, N=33.

#### Designed Online Communities

Similar prerequisites were identified in all of the designed community papers (Table 2) since functionalities were tailored according to the type of intervention being studied; the directions were (1) developing patients’ understanding of when to seek consultation (to become better informed); (2) providing inspiration through other patients’ stories in order to boost self-confidence in self-management of health condition (coping with emotional burden and networking with peers); (3) being equally involved in decision-making processes (to become better informed and absorb and reflect on information given by health care). An additional intervention purpose listed in all 5 community papers was how to expand research or health care services further in order to adapt to patients’ needs (5/33, 15%) [65-69].

The differences for the designed communities in comparison with the established communities were that the prerequisites were given by health care professionals or researchers, by informing patients during recruitment about the purpose of usage, which led to patients’ participation in the study, thus potential engagement and activation of health through the online community. Hence, the health care professionals were the leading part in the patient-provider relationship, since the recruitment was determined by the professionals’ confidence in the individual patient’s way of improving self-management through usage of community. However, during later phases of use, it was the patient who had the confidence in their ability, based on inspiration from other patients’ support in a particular online community.

#### Patient Activation and Engagement

Patient activation is described as the phase when patients act through knowledge gained, and create intermediate goals in order to improve their health condition. Additionally, it is presented as the phase in which patients know where to acquire knowledge and what they need to do to receive it [5,6]. Patient activation is often considered to be intertwined with patient engagement, which is defined as the patient’s motivation for improving health condition through a collaborative relationship with health care professionals. To motivate patients, health care professionals need to make patients aware of care processes, which is thereby the first step required to create good conditions for patient involvement and participation [5,6].

#### Established and Undefined Online Communities

In the established and unspecified online community papers, patient activation and patient engagement were analyzed as an integrated process that was supported by patient peers and not by health care professionals. This integrated process was shown by the use of the online communities—how patients searched and absorbed needed information. Thus, this generated how open patients were to (1) change health habits, (2) wanting to help others in the online community, or (3) wanting to prepare for upcoming care consultation with health care professionals (Table 3). The context behind what was generated as motivation is presented in Table 3.

**Table 3:** The integrated processes and context of motivation in established and undefined community papers (N=28/33).

Characteristic			Established online community				Undefined online community		
			Papers, n (%)^a^		References		Papers, n (%)^a^		References
Total papers (n=28)			21 (64)		[37-57]		7 (21)		[58-64]
**The integrated process**									
	Change health habits	8 (24)		[37,40,45-47,51,56,57]		5 (15)		[58-60,63,64]	
	Helping others	14 (42)		[38-47,50,51,55,57]		4 (12)		[58-60,64]	
	Prepare for upcoming care consultation with health care professionals	8 (24)		[39,40,44,46,49,52,56,57]		5 (15)		[46,58,59,61,62]	
**Context behind motivation**									
	Take control over health	19 (58)		[37-40,43-57]		7 (21)		[58-64]	
	Improve ability and conditions for patient involvement	10 (30)		[37,40,43,44,46-49,54,56]		5 (15)		[59-62,64]	
	Heal emotionally	14 (42)		[38-47,50,51,55,57]		4 (12)		[58-60,64]	

^a^All percentages refer to the total number of papers, N=33.

#### Designed Online Communities

In the designed online community papers, patient activation and patient engagement were analyzed as separate processes. The role of online community support for patient activation was to create independence and was presented as patients taking part in other patients' narratives and discussion with patient peers. In 2 out of the 5 papers (2/33, 6%), it was described as reasons for patients to be able to construct individual goals that were relevant to the individual situation and presented as a basis for the decision-making process during health consultations [65,67]. Identification of patient engagement within 3 of 5 designed community papers (3/33, 9%) was analyzed as patients’ motivation to create good conditions for collaboration with health care professionals to understand how the collaboration could generate better health outcomes [65,67,68].

#### Additional Measurements of Patient Engagement

Patient engagement was also studied by and presented as measuring how active patients were in an online community in the form of number of visits, time spent on the online community, or whether patients had contributed to content or not. These measures were used in one designed community paper (1/33, 3%) [68] and in 5 established community papers (5/33, 15%). The established community papers did not focus on studying patient engagement but used patient engagement measures such as those previously mentioned, in order to study their selected concept of patient empowerment (Table 1) [38,39,41,42,53].

#### Patient Involvement and Participation

Patient involvement is presented as (1) an advanced phase of patient engagement through patients’ awareness of the patient role within different care processes, which thus contributes to a collaborative relationship with health care professionals [6], or (2) health care providers’ prerequisites to include the patient during consultation as a first step for a collaborative relationship, which will later lead to the patient being the one who determines the prerequisites for consultation and decision making. This latter phase is presented as *patient participation* by [5] and *patient involvement* by [6].

#### Established and Undefined Online Communities

The analysis of patient involvement and participation was identified through the outcome of using online communities for all papers, no matter what type or initiation. In established and unspecified online communities, the outcome was that patients experienced (1) increased participation during health care consultation; (2) awareness of roles, such as when and how to contact and gain better outcomes from consultation; and (3) becoming more informed and having up-to-date knowledge about treatments, care process, and control of emotional management of the condition, which indicated an increased level of self-care (Table 4).

**Table 4:** The outcome of patient involvement and participation in established and undefined communities.

Characteristic		Established online community		Undefined online community	
		Papers, n (%)^a^	References	Papers, n (%)^a^	References
Total papers (n=28)		21 (64)	[37-57]	7 (21)	[58-64]
**Outcome**					
	Increased participation during consultations	10 (30)	[37,39,40,44-47,51,54,57]	4 (12)	[58-60,64]
	Awareness of care trajectory and how to gain better outcomes from consultation	10 (30)	[37,39,40,44-47,51,54,57]	4 (12)	[58-60,64]
	Increased level of self-care	20 (61)	[37-52,54-57]	7 (21)	[58-64]
**Response by health care professionals**					
	Positive	3 (9)	[45,48,49]	2 (6)	[58,59]
	Negative	7 (21)	[43-45,48,49,55,57]	2 (6)	[58,59]

^a^All percentages refer to the total number of papers, N=33.

#### Response by Health Care

In 9 out of 33 established or undefined community papers (27%), patients perceived themselves as being more up-to-date via online communities than they felt health care professionals were [40,43,44,46,49-51,58,64]. In another 9 established or undefined community papers (27%), this is described as a positive response by health care professionals, and sometimes, as the opposite [43-45,48,49,55,57-59] (Table 4). If health care professionals had a positive response to usage, patients often experienced themselves as being increasingly involved and having a collaborative relationship with health care professionals. Consequently, this resulted in patients experiencing better navigation in online communities, which affected how to incorporate information that was relevant to their individual situation. If health care professionals had a negative response toward patients’ usage of online communities, the consequence was often described as patients’ experiencing not being involved during consultation. Instead, the responsibility was all in the hands of the health care professional and was described as being not satisfying for patients (7/33, 21%) [43,44,48,49,55,58,59]. In 4 papers (12%), this was described as a reason for asking patient peers instead of health care professionals for consultation [43,44,55,57], or decreasing contact with health care professionals (3/33, 9%) [43,44,57]. If patients did not experience involvement during the first consultation, it affected whether patients chose to share their experiences of using online communities with health care professionals (5/33, 15%) [45,48,49,58,59].

#### Designed Online Community Papers

In the designed community papers, the outcome from patient involvement or participation was oriented toward leadership. At first as the health care professionals who determined conditions for treatment (3/33, 9%) [65,67,68], while in later steps when patients had more experience using the online community, the outcomes were that patients determined the conditions—how much patients decided to participate during consultation [67]. In one paper [67], the later steps are described as both positive and negative. The positive outcomes were that patients had more understanding of individual responsibility for health conditions, thus were more self-reliant on management and became more involved during health care consultations. The negative outcomes were that many health care professionals experienced pressure to be available online 24/7, in order to respond to patient contact inquiries. The contact inquiries were mostly regarding turmoil that had emerged during patients’ use of the online community. There was a mismatch between the patients' needs and the time the health care professionals had for this type of work, and the health care professionals experienced that some patients did not consider professionals’ life outside work or understand that they had other patients to care for, and therefore, had limited time.

## Discussion

### Summary

This systematic review’s objective research question was “In what ways can participation in online communities support patient empowerment?” The findings indicated that participation in online communities, regardless of type, can be seen as a complementary resource to traditional health care, since communities helped patients get more out of the consultation with health care professionals by understanding when to contact or getting an insight from peers (into the whole care trajectory and what to expect at different phases). Therefore, online communities supported patient empowerment by helping the patients become engaged and have the possibility of being equal contributors in the patient-provider relationship [5,6,10]. Additionally, participation in online communities supported patients in healing the emotional wounds of a diagnosis or handling negative experiences of their care trajectory. The emotional and personal experiences seemed to be an essential factor behind patients becoming empowered, thus an online community was a space for dealing with these types of experiences. These findings are relevant since they indicate that the progression to self-care must include personal elements and spaces for dealing with diagnosis [73-75]. This seems to be a limited service given by health care, according to the patient needs identified in the papers that were included.

Even if relevant, the way online communities specifically support patient empowerment is complex and dependent on patients’ levels of health literacy and previous online community experiences. Therefore, we (1) discuss different types of empowerment identified; (2) present limitations with the papers that were included, and simultaneously, give suggestions for future research; (3) propose a framework that can be used for understanding or evaluating in which way participation in online communities could support patients empowerment levels and potential progression, and (4) present limitations in conducting this systematic review, how it might have affected the inclusion of papers and the findings, and recommendations for future research concerning how to improve future conduct of systematic reviews.

### Different Types of Empowerment

One of this systematic review's contributions is identifying how participation in online communities supported patient empowerment as both a process and an outcome, which echoes results from and ideas in previous research [3,70,72,76]. The processes and outcomes that are supported depend on initiative and motivation to use online communities; hence there is an importance in unpacking the underlying factors for the way online communities supported patient empowerment. This is similar to identifications made by previous research regarding defining or evaluating patient empowerment in a traditional care trajectory [5,6,9], but also through online communities and other eHealth technologies [3,70,72,76,77].

Patient empowerment processes are often defined as continuously taking part in various forms of empowerment [6]. The systematic review confirms this and shows that these processes are identified through patient enablement, activation and engagement, via support from patient peers, and do not explicitly involve health care professionals. These processes include becoming better informed, receiving and giving emotional support by sharing relatable experiences of living with the diagnosis, helping others, and networking—which are best enhanced by peers. The outcomes identified through patient activation or involvement and participation were also considered suitable to be supported by patient peers rather than by health care professionals. The outcomes of becoming more active included patients’ experiences of being better informed, thus affected taking an increasingly leading role during health consultations and was seen through independence shown in self-care, adherence to treatment, acceptance of the diagnostic situation, feelings of control, emotional health, and self-efficacy. These examples are in line with those in previous research [3,70,72,76,77]; however, our paper adds an in-depth understanding of the differences in what these different concepts entail and how they are interrelated. In some papers that were included, the overall outcomes were discussed as leading to collective empowerment, where the aim was to gain collective knowledge within a community in order to make changes to health care services, systems, or ways of financing health care. Therefore, this presents opportunities for online communities to support patient progress to specific levels of empowerment [78]. Collective empowerment through online communities has been a recurring topic of interest in previous research regarding empowerment of employee, individual or consumer motivation to create change within an organization, community or business [79-81] but is not explored as often in online communities for health care purposes [78].

### Limitations in the Selected Papers and Future Research

Suggestions for future work is through 3 main areas of interest that concerns the identified limitations of the papers that were included: (1) study recruitment methods, (2) involvement of health care perspective, and (3) measurement of patient empowerment.

#### Study Recruitment Methods

Most papers that studied established online communities recruited respondents via sharing links in online communities, such as a questionnaire or interview request [38,39,41-43,45-47,50-52,56]. Consequently, this resulted in mostly positive respondents since they already used the online community enough to see the link. There were thereby both issues of sampling and of demographic characteristics (ie, well-educated, with good experience of using the internet, and high health literacy) that resulted; however, papers discussed these as limitations. Therefore, more diverse recruitment methods and a wider selection of respondents is crucial for future research.

##### Skills Required When Using Online Communities

Despite the limitations regarding recruitment, the findings could be perceived as patients’ needing previous experience and skills using online communities in order for the patient to be empowered and fulfill the purpose behind usage. There were patients that had negative experiences of using online communities if their needs were not met as expected. Another important factor was if the amount of information became too much to handle and was based on the individual phase of the diagnostic journey. Usually, the success of online communities depends on members’ previous experiences with using internet-related services, the functionality of technology, and their motivation for becoming a member in the first place [21-23]. Therefore, it is important for future research to consider a variety of respondents in order to understand how knowledge and design in online communities could be adapted and evaluated to those who have less prior experience in order for online communities to be beneficial and in order to reduce the risk of digital divide [3,18].

##### Involvement of a Health Care Perspective

There is a gap in involving a health care perspective. By this we refer to involvement such as integrating health care professionals’ views in order to understand how patients have been empowered when participating in online communities and to understand how online communities can be used and acknowledged as a complement to traditional health care. In all papers that were included, there were recommendations for professionals to be better involved and positive toward patient usage. However, only one paper [48] explicitly described how this could be managed. In order for professionals to see the potential of online communities or follow recommendations from research, there need to be strategies on how the use of established and designed online communities should be practiced in daily work and service routine [74,82].

Another limitation was difficulty in identifying level of participation of health care professionals contributing to content in online communities and how it affected patients. Patients and professionals are considered complementary actors of the patient empowerment experience [5,6]; therefore, the health care professionals’ perspective in relation to patient usage should be more involved and highlighted in online community research. This limitation identified in the papers that were included might depend on the conduct of search strategy and inclusion and exclusion criteria of this systematic review (Table S1, Multimedia Appendix 2).

##### Measurement of Patient Empowerment

Measurement and definition of patient empowerment were also a limitation in selected papers. In inductive approach papers, patient empowerment was mainly measured through respondents stating they felt empowerment, while in deductive papers through measurements. There were many individual adaptations of measurement tools, which made it difficult to evaluate the quality of studies [19]. These limitations depend on patient empowerment as a concept that has many different definitions and interpretations depending on context and diagnosis [5,6,10,67,72-74]. Another reason can be that there are few validated analytical frameworks that can be used when evaluating health-related effects in online health communities [19]. Therefore, we propose a revised framework that aims to evaluate different levels of patient empowerment, and progression made through online communities. This framework was developed during the analysis process.

### Framework of Patient Empowerment Levels

Based on the findings in this systematic review in relation to the limitations identified, we propose a revised framework for empowerment consisting of hierarchical levels (Table 5). These hierarchical levels can be used to identify in which way an online community can support patient empowerment processes or to construct trajectories of progress based on the patients’ needs and where the patients are on their journey. Additionally, the framework can give guidance in how to methodically identify hierarchical levels of patient empowerment. By hierarchical levels we mean levels that can describe phases that patients are currently in, which can make it easier to evaluate paths for progression. There is a general consensus regarding progression as part of the empowerment concept, regardless of which concepts of patient empowerment are being used to discuss it [1,6,7,10,79-81]. Therefore, when discussing online communities in relation to patient empowerment, progression within specific phases is an important aspect. The progression affects the way the patients perceive their own empowerment and how susceptible they are to motivation and effects of self-care, their feelings of control, and the way they conduct contact with health care professionals. It is also important to keep in mind that not all patients want or have the ability to progress.

Additionally, the level of empowerment also affects how patients use online communities as support in the process of progression and how they support others, which in turn affects the collective empowerment (Table 5). Identifying where a patient is in the progression process is therefore an important aspect and the framework can be used to do so. However, it is important to keep in mind that people who have satisfied their informational and support needs may choose to leave the community or do not want to continue their empowerment progression by becoming producers or patient mentors in the online community. The reason might be that they believe to have completed their diagnostic journey, thus do not want to be reminded of their previous situation [83]. Another aspect to consider before using this type of framework is the nonlinearity of potential empowerment progression, that can happen if, for example, a new diagnosis or something else happens that affect management of condition and trajectory. Another example to consider is that several levels could occur simultaneously and might depend on the structure of the online community [21-23]. No matter what, the most important aspect to keep in mind is that not all patients want or have the ability to progress; therefore, we recommend that others evaluate this framework with caution.

**Table 5:** The proposed hierarchical framework of patient empowerment levels.

Hierarchical level of patient empowerment	Definition of level
Level 1: Motivated patient	The motivated patient is motivated to adhere to treatment and information given by the professional but lets the professional take the leading role within health care consultation.
Level 2: Self-cared patient	The self-cared patient takes control over disease and seeks information and knowledge that will help improve self-management or takes a leading role in health consultation. The patient uses the online community in order to get a second opinion or support potential void of information or knowledge from health care, regarding emotional or social aspects of living with the disease. At this level, the patient is thus more driven and uses online communities as complements as to traditional health care and has taken ownership of the disease.
Level 3: Producing patient	The producing patient means that the patient is not just a passive consumer of care since the patient wants to help others by sharing their experiences with disease and health care process. The context behind helping others might depend on the patient wanting to learn, and simultaneously gain status and satisfaction, in order to improve emotional or mental health. Additionally, the context of learning and gaining status or satisfaction and how it improves emotional or mental health could be an outcome of helping others.
Level 4: Patient activist	Collective empowerment is considered to be the patient having the aim of informing or helping others in order to develop or change policies and awareness in health care. Patients’ experiences become evidence-based and used by health care professionals.

### Limitations of This Work

#### Way of Reporting Included Papers

The main characteristics of papers that were included were individually reported (Multimedia Appendix 6). However, full descriptions of details such as country of origin, the sample size of respondents, and the online community's domain were not always presented. Papers differed with their transparency, which made it difficult to report consistently and took a lot of time. Hence, we decided to exclude categories typically reported in systematic reviews, which may limit the transparency and quality of our findings. Therefore, we recommend future research to divide the work of systematic review into phases of looking into specific details of the papers that were included and reporting them in a particular time period in order to make it time-efficient and simultaneously maintain the quality of work that is expected of systematic reviews [24,34,84].

#### No Quality Rating

It could be argued that it is standard to use quality rating of papers that were included in systematic reviews to evaluate risk of bias and how to evaluate the validity of the findings identified [24,84]. The reason for not including quality rating in this systematic review was based on wanting to focus on how to visualize the analysis of patient empowerment in order to make potential contribution of making the concept clear when put into specific context [5,6,9]. Additionally, it was difficult to evaluate which type of quality rating standard should be used since the papers that were included varied (in measuring empowerment and context of studies; Multimedia Appendix 6). This difficulty depended on the objective, time limitation, and not following a linear process in planning and conducting the systematic review. Therefore, we recommend future research to carefully structure the planning phase of doing systematic reviews in order to follow the principles of the PRISMA statement and rules of conduct when doing high-quality systematic reviews.

### Conclusion and Future of Patient Empowerment in Online Communities

This systematic review shows in which ways participation in online communities could support patient empowerment. The main findings indicated that online communities supported patient empowerment in the way of meeting emotional need of handling condition and the possibility of patients becoming equal contributors to the patient-provider relationship. An additional finding was that online communities supported both process and outcomes of patient empowerment. The main contribution of this systematic review is a framework and conceptualization of how patient empowerment in online communities can be understood, evaluated, and designed for empowerment progression and support. Based on identification of main findings, we suggest that future work look specifically toward 3 main areas of interest: (1) study recruitment methods; (2) involvement of a health care perspective; and (3) measurement of patient empowerment. Based on all suggestions, we propose that our framework can be used to evaluate different levels of patient empowerment and progression through online communities.

## Appendix

Multimedia Appendix 1 Report of the PRISMA (Preferred Reporting Items for Systematic Reviews and Meta-Analyses) guidelines.

Multimedia Appendix 2 Inclusion and exclusion criteria.

Multimedia Appendix 3 Database searches.

Multimedia Appendix 4 Analytical framework of patient empowerment.

Multimedia Appendix 5 Excluded papers.

Multimedia Appendix 6 General characteristics of selected papers.

## Abbreviations

PRISMA: Preferred Reporting Items for Systematic Reviews and Meta-Analyses
