# Supplementary material for: Online Communities as a Driver for Patient Empowerment: Systematic Review
Source: J Med Internet Res. 2021 Feb 9;23(2):e19910. doi: 10.2196/19910 (PMC7902187; doi:10.2196/19910)
Supplement: Multimedia Appendix 2 [file jmir_v23i2e19910_app2.docx]

Table 1. First Selection Process Criteria

| First Selection process | | Selection Criteria | |
| --- | --- | --- | --- |
|  |  | Description of Criteria | Reason |
|  |  |  |  |
| **Inclusion criteria** | |  |  |
|  | Patient Empowerment Perspective | Papers needed to include the perspective of patient empowerment or the related concepts, Patient enablement, Patient activation, Patient engagement, Patient involvement, or Patient participation. Although, the different concepts do not explicitly need to be written out, but the contribution of the study should relate to one of these concepts. | As stated in the method section of the manuscript, we used the work by [1,2] for the analysis presented in results. The reason was based on to better identify what is considered to be patient empowerment or how patient empowerment could be described. |
|  | Online Community Perspective | Papers needed to be in the format of text-based online discussion forums. If results contained social media or other online communities in relation to patient empowerment, this was a including criteria as well. | The reason for having the criteria was due to our research interest for the systematic review of studying this format of online communities |
|  | Patient perspective | Papers needed to be centered to the patient usage of online community, and how their usage effect/contribute to level of patient empowerment. Although, if an article contained a perspective of involving care provider/personnel in the patient perspective, this type of articles would be included as well. | The reason for this type of inclusion was based on following the essence of patient empowerment that is centered to the patient. Additionally, the interest to involve papers that also included professionals’ usage, was based on to see how potential patient-provider relationship in online communities would manifest and affect the structure in the community and patient satisfaction/dissatisfaction. |
| **Exclusion criteria** | |  |  |
|  | Philosophical perspective | This meant that papers that were focused on patient empowerment and the usage of online communities from a philosophical interest were excluded. This meant that papers that lacked empirical anchoring and were instead conceptual papers were excluded. | The reason for having this exclusion criteria was based on the wish for contribution with the systematic review, which is to clarify patient empowerment when the concept is studied in a particular context. Thus, we believed would be better clarified if papers would have empirical anchoring than a philosophical interest. |
|  | Only Professional perspective | This meant that papers that were centered only to healthcare professionals’ usage of online communities and their perception of patient empowerment were excluded. | The reason is based on to better identify answers to the systematic review’s objective and research question in relation to the essence of the patient empowerment concept of being centered to patient usage. |
|  | Did not contain any Patient empowerment perspective or Online community perspective | This meant that even if papers only presented either patient empowerment or online community alone, and not in combination, papers were excluded. | The reason is based on to better identify answers to the systematic review’s objective and research question. |

Table 2. Second Selection Process Criteria

| Second Selection process | | Selection Criteria | |
| --- | --- | --- | --- |
|  |  | Description of Criteria | Reason |
|  |  |  |  |
| **Inclusion criteria** | |  |  |
|  | The same as inclusion criteria as in First Selection process | See Table 1 in this Appendix. | See Table 1 in this Appendix. |
|  | Evidence of Patient Empowerment | Patient empowerment needs to be discussed or presented throughout references, and have some sort of description of how the authors of papers measured, or reasons for claiming patient empowerment in their results. | We noticed for instance, papers that mentioned patient empowerment only in the introduction, and not later throughout the paper or as part of results. Thus, created difficulties of remaining neutral and seeing the characteristics of the paper of what it actually was, without enforcing our own interpretation of how this could answer our research question - which would go against the purpose of doing a systematic- [3,4] or literature reviews [5].  Additionally, there were also papers were patient empowerment was presented only in the results but did not describe how conclusions of empowerment became emerged or was built-upon. Thus, created the same difficulties as already presented, but also that it would go against the aim for potentially contributing to strategic development with empirical validity. |
| **Exclusion criteria** | |  |  |
|  | Not finding full-text | For becoming included, papers needed to be in full-text. | The reason was based on getting full details of each paper and see if papers followed the decided inclusion and exclusion criteria. |
|  | Papers were not in English or in a native language | Papers needed to be in English or in Authors native language | To understand what we read. |
|  | The same as Exclusion criteria for First Selection process | See Table 1 in this Appendix. | See Table 1 in this Appendix. |
|  | Format other than articles and research papers | If a papers were in other format than article or research papers, the paper was excluded. | In the search strategy we had selected the criteria of format, but we noticed that some papers were e.g. books, reviews and research posters. The reason for exclusion of other formats was based on the time to conduct the systematic review, and potential difficulties of handling different formats during the review process [3,4]. |
|  | No Evidence of how Patient Empowerment was evidenced. | See the Inclusion criteria of Table 2. | See the Inclusion criteria of Table 2. |

**References**

1. Fumagalli LP, Radaelli G, Lettieri E, Bertele P, Masella C. Patient Empowerment and its neighbours: Clarifying the boundaries and their mutual relationships. *Health Policy.* 2015;119(3):384-394.

2. Palumbo R. *The bright side and the dark side of patient empowerment: Co-creation and co-destruction of value in the healthcare environment.* Springer; 2017.

3. Bryman A. *Social research methods.* Oxford university press; 2016.

4. Knopf JW. Doing a literature review. *PS: Political Science & Politics.* 2006;39(1):127-132.

5. Webster J, Watson RT. Analyzing the past to prepare for the future: Writing a literature review. *MIS quarterly.* 2002:xiii-xxiii.
