# Supplementary material for: Online Communities as a Driver for Patient Empowerment: Systematic Review
Source: J Med Internet Res. 2021 Feb 9;23(2):e19910. doi: 10.2196/19910 (PMC7902187; doi:10.2196/19910)
Supplement: Multimedia Appendix 3 [file jmir_v23i2e19910_app3.docx]

| **Database** | **Total number of articles** | **Total after duplicates removed** |
| --- | --- | --- |
| SCOPUS | 701 | 654 |
| PubMed | 406 | 358 |
| EBSCOHOST | 128 | 112 |
| ACM | 82 | 53 |
| Web of Science | 28 | 10 |
| **Total** | **1345** | **1187** |

The differences of searches and results from the selected databases.

***Disclaimers:***

- Search with **ACM** **Digital Library** were conducted manually for each combination of keywords e.g., “patient empowerment” AND online communit* – “patient empowerment” AND “online interpersonal communication” and so on. Unfortunately, the specific search strings for this database were not saved due to authors’ did not create an account before conduct of search. This was noticed months after the search was conducted.
- In order to retrieve search history results, some of the databases have not restored the actual data according to date of search (17 January 2019). Some of the saved searches only had the updated numbers of current dates results. Therefore, some numbers are not revealed in some tables below.

**SCOPUS**

| Date | Topic of search | Search string | Results |
| --- | --- | --- | --- |
| 17 January 2019 | PE search | ( ALL ( "patient empowerment" ) OR ALL ( "patient activation" ) OR ALL ( "patient enablement" ) OR ALL ( "patient engagement" ) OR ALL ( "patient involvement" ) OR ALL ( "patient participation" ) ) | 58,968 |
|  | OC search | ( ALL ( "online communit*" ) OR ALL ( "online peer-support" ) OR ALL ( "online interpersonal communication" ) OR ALL ( "online health communit*" ) OR ALL ( "online patient support" ) OR ALL ( "online social support" ) OR ALL ( "online peer-to-peer support" ) ) | 32,601 |
|  | PE & OC combo search | ( ( ALL ( "patient empowerment" ) OR ALL ( "patient activation" ) OR ALL ( "patient enablement" ) OR ALL ( "patient engagement" ) OR ALL ( "patient involvement" ) OR ALL ( "patient participation" ) ) ) AND ( ( ALL ( "online communit*" ) OR ALL ( "online peer-support" ) OR ALL ( "online interpersonal communication" ) OR ALL ( "online health communit*" ) OR ALL ( "online patient support" ) OR ALL ( "online social support" ) OR ALL ( "online peer-to-peer support" ) ) ) | **701** |

**EBSCO (Cinahal & Medline)**

| **Date** | **Topic of search** | **Search string** | **Results** |
| --- | --- | --- | --- |
| 17 January 2019 | PE search | TX "patient empowerment" OR TX "patient activation" OR TX "patient enablement" OR TX "patient engagement" OR TX "patient involvement" OR TX "patient participation" |  |
|  | OC search | TX "online communit*" OR TX "online peer-support" OR TX "online interpersonal communication" OR TX "online health communit*" OR TX "online patient support" OR TX "online social support" OR TX "online peer-to-peer support" |  |
|  | PE & OC combo search | S1 AND S2 | **128** |

**PubMed**

| Date | Topic of search | Search string | Results |
| --- | --- | --- | --- |
| 17 January 2019 | PE & OC combo search | ((((((( "patient empowerment" ) OR ALL ( "patient activation" ) OR ALL ( "patient enablement" ) OR ALL ( "patient engagement" ) OR ALL ( "patient involvement" ) OR ALL ( "patient participation" ) ) ) AND ( ( ALL ( "online communit*" ) OR ALL ( "online peer-support" ) OR ALL ( "online interpersonal communication" ) OR ALL ( "online health communit*" ) OR ALL ( "online patient support" ) OR ALL ( "online social support" ) OR ALL ( "online peer-to-peer support" ) ) ) | 406 |

**Web of Science**

| Date | Topic of search | Search string | Results |
| --- | --- | --- | --- |
| 17 January 2019 | PE search | **ALL FIELDS:** ("patient empowerment") *OR* **ALL FIELDS:** ("patient activation") *OR* **ALL FIELDS:** ("patient enablement") *OR* **ALL FIELDS:** ("patient engagement") *OR* **ALL FIELDS:** ("patient involvement") *OR* **ALL FIELDS:** ("patient participation")  *DocType=All document types; Language=All languages;* |  |
|  | OC search | **ALL FIELDS:** ("online communit*") *OR* **ALL FIELDS:** ("online peer-support") *OR* **ALL FIELDS:** ("online interpersonal communication") *OR* **ALL FIELDS:** ("online health communit*") *OR* **ALL FIELDS:**("online patient support") *OR* **ALL FIELDS:** ("online social support") *OR* **ALL FIELDS:** ("online peer-to-peer support")  *DocType=All document types; Language=All languages;* |  |
|  | PE & OC combo search | #2 AND #1  *DocType=All document types; Language=All languages;* | **28** |
