# Supplementary material for: Online Communities as a Driver for Patient Empowerment: Systematic Review
Source: J Med Internet Res. 2021 Feb 9;23(2):e19910. doi: 10.2196/19910 (PMC7902187; doi:10.2196/19910)
Supplement: Multimedia Appendix 4 [file jmir_v23i2e19910_app4.docx]

*Patient Enablement Patient Activation Patient Engagement Patient Involvement Patient Participation*

**Fumagalli et al. (2015)** Focus on the possibilities and

prerequisites healthcare gives the

patient in order to become engaged and active in her/his own health, understanding and handling of diagnosis.

When the patient understands and has knowledge to manage his/her diagnosis. In order to get this understanding/knowl­edge of handling, the patient is aware of what he/she needs to do. Thus, the patient creates intermediate goals that should have the ability to improve health/handling of the diagnosis. The patient becomes “activated.”

Patient's own motivation for improved health/ management of health.

Additionally, also the motivation for having a relationship with healthcare professionals in order to create a joint participation prior to healthcare visit/journey. The healthcare professional must create measures that will keep the patient engaged.

Focus on what prerequisites healthcare has to include the patient during care visit. For the first step, the healthcare professional is the person leading the care visit, but then it is intended that the patient via **“patient participation”** should become the leader during the care visit. There are two different types of involvement: patient determined and professional determined.

The patient becomes the leading party in the care visit. Collaboration between healthcare professional and the patient.

**Palumbo (2017)** Focus on the patient's confidence

in the ability to improve their

knowledge, experiences and management of diagnosis and healthcare professional-patient relationship

When the patient acts through the knowledge he/she has gained, in order to make changes that will improve their well-being. Motivation and awareness are the foundation and creation of interim goals for the patient to manage his/her own health.

The patient's motivation to create meaningful relationships with healthcare professionals in order to improve management of his/her own health. In order for the patient to be motivated, it is the responsibility of the healthcare professional to make the patient aware of their role in creating participation between the healthcare professional and the patient.

Defined as an advanced stage of patient engagement. Focus on the patient's understanding of his/her role in various care processes, which contributes to a collaborative relationship between the healthcare professional and the patient.

Seems to be included in the definition of patient involvement. There is no specific definition of patient participation by Palumbo (2017).

**Similarities/Differences** *Similarities:* Focus on the

prerequisites for starting a patient

empowerment process for a patient

*Differences:* Various actors who will take responsibility within this phase/concept

*Similarities:* The same kind of definitions, but differently formulated regarding choice of words/sentences. Specific focus on diagnosis in this phase/concept.

*Differences:* More clarity by Palumbo (2017), since it is described that the act of the patient will generate independence in handling the diagnosis. This can be inferred in Fumagalli et al. (2015).

*Similarities:* Relatively similar definitions. The aim of this phase is to be the first phase of patient involvement/ participation. Healthcare professionals have the main responsibility in this phase.

*Differences:* None, except different ways of formulating words/sentences.

*Similarities*: The patient is better aware of their role in the care process.

*Differences:* Palumbo (2017) seems to make a linked definition similar to the definition by Fumagalli et al. (2015) of both patient involvement and patient participation. Seems to be more responsible for the patient in Palumbo's definition of involvement than in the definition by Fumagalli et al. (2015).

*Similarities:* See the previous comment regarding similarities and differences for patient involvement.

*Differences:* Patient participation is not defined/mentioned in Palumbo (2017).
